# BUILDING BRIDGES, CATALYZING RESEARCH, EMPOWERING ALL AGES: A CHAT WITH NIA SENIOR LEADERSHIP

**DOI:** 10.1093/geroni/igad104.0831

**Published:** 2023-12-21

**Authors:** Amy Kelley, Amy Kelley

## Abstract

The National Institute on Aging (NIA) at the National Institutes of Health, Department of Health and Human Services, is the federally designated lead agency on aging research and supports research to understand the nature of aging and to extend the healthy, active years of life. Over the last decade, NIA’s budget has increased substantially. In keeping with NIA’s scientific mission, funding includes significant investments in Alzheimer’s disease (AD) and AD-related dementias research, as well as in non-AD research. This symposium will provide a forum for exploration of the implications of the NIA budget for the general research community. NIA’s senior staff will discuss research priorities and programs supported by the Institute. A question-and-answer session will follow these remarks on current funding and future priorities and research directions of NIA.

